# Leaders’ experiences of embedding a simulation-based education programme in a teaching hospital: an interview study informed by normalisation process theory

**DOI:** 10.1186/s41077-024-00294-3

**Published:** 2024-05-20

**Authors:** Rebecca A. Szabo, Elizabeth Molloy, Kara J. Allen, Jillian Francis, David Story

**Affiliations:** 1 Department of Critical Care, Melbourne Medical School, The University of Melbourne, Melbourne, VIC 3052 Australia; 2 Department of Obstetrics and Gynaecology, Melbourne Medical School, The University of Melbourne, Melbourne, Australia; 3 Department of Medical Education, Melbourne Medical School, The University of Melbourne, Melbourne, Australia; 4 Gandel Simulation Service, The Royal Women’s Hospital, Parkville, VIC Australia; 5 Faculty of Medicine, Dentistry and Health Sciences, The University of Melbourne, Parkville, VIC Australia; 6 School of Health Sciences, The University of Melbourne, Parkville, VIC Australia; 7 Department of Health Services Research, Peter MacCallum Cancer Centre, 305 Grattan St, Melbourne, VIC 3000 Australia; 8 Sir Peter MacCallum Department of Oncology, The University of Melbourne, Melbourne, VIC 3010 Australia; 9 Centre for Implementation Research, Ottawa Hospital Research Institute — General Campus, 501 Smyth Road, Ottawa, ON K1H 8L6 Canada

**Keywords:** Simulation-based education, Sustainability, Normalisation, Leadership, Change management, Implementation

## Abstract

There is limited research on the experiences of people in working to embed, integrate and sustain simulation programmes. This interview-based study explored leaders’ experiences of normalising a simulation-based education programme in a teaching hospital. Fourteen known simulation leaders across Australia and North America were interviewed. Semi-structured interviews were analysed using reflexive thematic analysis sensitised by normalisation process theory, an implementation science theory which defines ‘normal’ as something being embedded, integrated and sustained. We used a combined social and experiential constructivist approach. Four themes were generated from the data: (1) Leadership, (2) business startup mindset, (3) poor understanding of simulation undermines normalisation and (4) tension of competing objectives. These themes were interlinked and represented how leaders experienced the process of normalising simulation. There was a focus on the relationships that influence decision-making of simulation leaders and organisational buy-in, such that what started as a discrete programme becomes part of normal hospital operations. The discourse of ‘survival’ was strong, and this indicated that simulation being normal or embedded and sustained was still more a goal than a reality. The concept of being like a ‘business startup’ was regarded as significant as was the feature of leadership and how simulation leaders influenced organisational change. Participants spoke of trying to normalise simulation for patient safety, but there was also a strong sense that they needed to be agile and innovative and that this status is implied when simulation is not yet ‘normal’. Leadership, change management and entrepreneurship in addition to implementation science may all contribute towards understanding how to embed, integrate and sustain simulation in teaching hospitals without losing responsiveness. Further research on how all stakeholders view simulation as a normal part of a teaching hospital is warranted, including simulation participants, quality and safety teams and hospital executives. This study has highlighted that a shared understanding of the purpose and breadth of simulation is a prerequisite for embedding and sustaining simulation. An approach of marketing simulation beyond simulation-based education as a patient safety and systems improvement mindset, not just a technique nor technology, may assist towards simulation being sustainably embedded within teaching hospitals.

## Introduction

Simulation should be embedded as a normal part of healthcare to improve patient safety and outcomes, as normal as an operating theatre or emergency department. Actual implementation remains variable despite strong evidence to support simulation-based education (SBE) as an effective pedagogy [1–4]. The global growth of simulation has been ‘sporadic and unequal’ [5, 6], and many simulation programmes in hospitals can fail to reach their full potential [7].

With considerable evidence for SBE as an effective pedagogy, there is a need for a focus on widespread implementation [2, 8–12]. However, few empirical studies have explored the implementation of SBE broadly [6]. The impetus for this exploratory study was to learn from leaders who have successfully embedded SBE programmes — or failed and learned important lessons. We were also keen to understand what may further influence sustained change to fully integrate simulation for ‘safety, quality and — where it does not conflict with these goals — for efficiency’ ([8] p.i2).

Understanding and incorporating implementation science and practice may help us move past this variation in SBE globally. Implementation refers to the process of putting a plan or decision into place. Implementation science seeks to provide evidence about how to accelerate the processes of change to facilitate the incorporation of evidence-based approaches into practice and reduce the time lag from discovery to practice [13].

Much effort is required to operationalise novel practices, such as simulation, within a complex adaptive system and needs to be informed by evidenced-based approaches and theories [9, 14]. Normalisation process theory (NPT) is one implementation science theory which defines normalisation as an intervention being both embedded and integrated into an organisation [15]. NPT provides a flexible science framework that can be used to direct attention to understand how interventions are implemented, embedded and sustained. It has been widely used for implementation and evaluation of both healthcare and healthcare professions education interventions [15].

While limited, studies of implementing SBE have identified enablers and barriers including learners’ needs, educators’ vision, funding, staff, space, equipment, buy-in, governance and faculty development [16–18]. In 2020, Ferguson et al. explored the implementation of SBE in undergraduate healthcare professional programmes in the north of England using NPT [6]. They found that a lack of understanding, supporting infrastructure, leadership and strategy impeded full realisation of the benefits of SBE. Given the complexity of implementation [19], we were keen to explore how this applies in teaching hospital settings, for all staff and students. In 2023, Shah et al. explored integrating simulation into surgical training using NPT for secondary data analysis [20]. They found that distributed leadership attracted wide engagement and promoted successful normalisation [20].

We therefore chose to use NPT to sensitise our analysis in seeking to understand how SBE programmes were embedded sustainably in teaching hospitals NPT. The term ‘normal’ is used throughout to mean embedded and integrated or sustained such that a programme becomes ‘routine business’. NPT proposes that four constructs facilitate normalisation: coherence, cognitive participation, collective action and reflexive monitoring [15]. Each of these constructs has four components [15]. The components of NPT are further described in detail in Table 1 and are expanded upon in discussion as they relate to our study findings.

**Table 1:** NPT constructs and components as described by Finch et al. [21] Reproduced with permission from Huddlestone et al. [22]

**NPT construct**	**Coherence**	**Cognitive participation**	**Collective action**	**Reflexive monitoring**
	The process and work of sense making and understanding that individuals and organisations undertake that promote or inhibit the routine embedding of a practice	The process and work that individuals undertake to promote engagement with the new practice	The work done by individuals and organisations to enact the new practice	The work inherent to formal and informal appraisal of new practice, to enable assessment of advantages and disadvantages, developing users’ comprehension of the effects of a practice
**NPT components**	**Differentiation**	**Enrolment**	**Interactional workability**	**Systematisation**
	Do stakeholders see this as a new way working?	Do the stakeholders believe they are the correct people to drive forward the implementation?	Does the intervention make it easier or harder to complete tasks?	Will stakeholders be able to judge the effectiveness of the intervention?
	**Individual specification**	**Initiation**	**Skill set workability**	**Individual appraisal**
	Do individuals understand what tasks the intervention requires of them?	Are they willing and able to engage others in the implementation?	Do those implementing the intervention have the correct skills and training for the job?	How will individuals judge the effectiveness of the intervention?
	**Communal specification**	**Activation**	**Relational integration**	**Communal appraisal**
	Do all those involved agree about the purpose of the intervention?	Can stakeholders identify what tasks and activities are required to sustain the intervention?	Do those involved in the implementation have confidence in the new way of working?	How will stakeholders collectively judge the effectiveness of the intervention?
	**Internalisation**	**Legitimation**	**Contextual integration**	**Reconfiguration**
	Do all the stakeholders grasp the potential benefits and value of the intervention?	Do they believe it is appropriate for them to be involved in the intervention?	Do local and national resources and policies support the implementation?	Will stakeholders be able to modify the intervention based on evaluation and experience?

Further detail and NPT toolkit available at https://normalization-process-theory.northumbria.ac.uk/ [15]

Our conceptual approach underpinned by implementation science was also complemented by literature on leadership and change management. This builds on existing findings that buy-in and leadership [6] are enablers and were influenced by the lead researcher (R. S.) creating the business case for a simulation programme. Change management is ‘the process of continually renewing an organisation’s direction, structure and capabilities to serve the ever-changing needs or internal and external customers’ ([23] p.66). While change management is primarily associated with the business world, it overlaps with many of the practices and theories of implementation science. Recent work by simulation scholars Eller et al. in 2023 has explored the organisational change needed for the implementation of three in situ simulation programmes.^22^ The inclusion of business and organisational literature with implementation science provides an interdisciplinary lens to contribute to a comprehensive understanding of the organisational change required for embedding SBE.

Our work sought to build on the work of others by exploring the research question: how is SBE normalised in a teaching hospital?

### Aim

The aim of this research was to understand healthcare simulation leaders’ experiences of normalising a simulation-based education (SBE) programme in their teaching hospital.

### Methodological positioning

#### Researcher positioning

The authors have a combination of clinical, education, research, theory and content expertise. The lead researcher, R. S., is an obstetrician/gynaecologist and medical educator with some research experience. She leads a simulation service, which was established in February 2020 based on a business case created from a previous smaller study [24]. D. S. is a specialist anaesthetist and experienced researcher with several leadership roles. J. F. is an implementation scientist with extensive experience and knowledge of implementation science frameworks and methods. E. M. is an experienced health professions educator and researcher with expertise in qualitative methodologies and experience in establishing interprofessional education programmes for health professions at universities and hospitals. K. A. is a specialist anaesthetist and simulation leader who has conducted research focused on leadership and change management.

We adopted a combined social and experiential constructivist worldview. Social constructivism describes the role of sociocultural factors on how humans learn. It is relevant for this study as the experience of SBE is likely to vary depending on the person, the location and culture, something that was recognised by Gaba in his future vision of simulation [8]. Experiential constructivism incorporates the lived experience and reflections of individuals, in this case, of simulation leaders and researchers R. S. and K. A. As R. S. has established and embedded a simulation service at a teaching hospital, she is inextricably part of this research such that reflexivity and the inclusion of her lived experience are integral to the process. K. A. is also part of this simulation service and has led another simulation service so contributed lived experience.

#### Conceptual framing

This combined social and experiential constructivist approach sits within a Big Q [25] qualitative research paradigm compared to a little q approach where qualitative methods are used but with a positivist or post-positivist stance [25]. A Big Q paradigm, first described by Kidder and Fine [26], describes research underpinned by qualitative research values such that it most equates with subjectivity and lived experience [25]. Clarke and Braun describe being a knowing researcher [25]. This aligns with Varpio et al. encouraging health professions education scholars to ‘avoid using qualitative terms uncritically and non-reflexively’ ([27] p.40) and our combined social and experiential constructivist worldview.

In the time since the data was collected, the world we live in has changed, noticeably in healthcare and teaching hospitals particularly due to the pandemic and impact on our workforce. Rather than this detracting from this study’s contribution to the literature, we feel this is a timely moment to contribute to a conversation on embedding sustainable SBE programmes, and our methodological orientation and theoretical approach aim to account for this. The lived experience of R. S. and K. A. in embedding a simulation service since February 2020 is significant and informed our theoretical approach to include implementation science as well as literature from the business world.

Based on our theoretical orientation, reflexive thematic analysis (RTA) [25] was selected as the analytical method. RTA is one method of thematic analysis and an evolution of what Braun and Clarke first described in 2006 [25, 28–30]. RTA acknowledges the researcher’s active role in analysing data and generating themes [25, 31] by recognising ways in which a researcher’s values, experiences, interests and sociocultural context inform the analysis [32]. Thus, reflexivity and a Big Q approach are integral for RTA practice and quality [30, 33]. While RTA is not a methodology in and of itself, Clarke has described it as ‘method-ish’ because of the philosophical Big Q framing [34, 35]. Our combined social and experiential constructivist approach intends to align with this because of the emphasis on researcher reflexivity.

The RTA focus on researcher reflexivity ‘makes “pure” induction impossible’ ([25] p.8). Instead, the researcher always brings a personal pre-existing lens to which they apply to analysis and are sensitised by both that and potentially other theoretical frameworks. Our analysis was further informed by NPT (Table 1) because of the central focus on understanding how SBE becomes embedded and integrated as part of ‘business as usual’.

As RTA was used within a Big Q paradigm, the structure of this paper does not follow the reporting structure traditionally used in quantitative and small q research. Instead, the results — or analysis, include literature as part of framing the analysis, situating and finding meaning in the data as well as researcher experience, consistent with RTA and our combined social experiential constructivist stance. This may feel more like a discussion to those unfamiliar with Big Q research, but this is part of RTA, actively generating and contextualising themes from the data, literature and lived experience and of being a knowing researcher [30].

## Methods

### Ethical considerations

Ethical approval for the study was obtained through the University of Melbourne Department of Medical Education Human Ethics Advisory Group (HEAG) (Project ID 1749545).

### Setting and sample

The sample size was based on pragmatic and theoretical rationales. The lead researcher (R. S.) chose to focus on Australia and North America. Australian sites were included to understand the researchers’ context. Although two Australian SBE programmes had been in place for just over 20 years at the start of this project, elsewhere in Australia, SBE programmes were far less established. In comparison, SBE programmes have been well-established in North America for several decades. It was felt exploring the research question in the USA would lead to important insights into what happens over time. A site in Canada was included due to its proximity to the USA and the health service being more comparable to Australia. Simulation programme directors and managers were identified as leaders best placed to answer the research question. Participants were simulation programme directors and managers who met the following inclusion criteria: (1) English speaking, (2) at least 5 years’ experience in simulation (preferably at a leadership level) and (3) based in a simulation centre or programme affiliated with a teaching hospital in Australia, Canada or the USA. At least 5 years’ experience was chosen based on the pragmatic rationale that individuals would have the experience and knowledge of both embedding and integrating a programme.

### Recruitment

Simulation programme directors and managers were identified through academic or institutional websites, peer-reviewed literature and snowball sampling [36]. For snowball sampling, participants were asked to recommend other SBE leaders. The primary institutional websites included the Australian Society for Simulation in Healthcare (ASSH) and the Society for Simulation in Healthcare (SSH) and simulation centre websites. Email addresses were obtained through these sites. Key informants were sent an email invitation with PDF versions of a plain language statement and consent form attached. Where snowball sampling was used, the referring leader provided an email introduction, and the lead researcher then followed up.

Twenty informants were invited to participate, 5 through snowball sampling. One did not respond; 19 replied and consented to participate. One participant with less than 5 years’ experience was excluded. Another was excluded as their simulation centre was not directly affiliated with a teaching hospital. The first interviews in each country, three in total, were analysed in phase 1 [24] and excluded from this study. Fourteen participants were included in this study, 9 based in Australia, 4 in the USA and 1 in Canada. Five participants had more than 15 years’ experience, six had 12–15 years’ experience, two had 8–11 years’ experience and one had 5–8 years’ experience.

### Data collection and process

Semi-structured interviews were conducted by R. S. in late 2017 and early 2018 based on an interview guide that had been pilot-tested. Participants were given the option of face to face, phone or video conference call. All included interviews were conducted face to face. An anonymous ID was created for each participant, and all data was stored in a password-protected secure format. Interviews were audio-recorded with consent using the mobile app RecUp (Irradiated Software, LLC^©^ 2017). Interview durations ranged from 40 to 90 min. A professional transcription service was used.

### Data analysis and reporting

Data analysis was conducted in 2022 and 2023. Data was analysed using RTA sensitised by NPT. Analysis followed the six RTA steps of (1) familiarisation; (2) coding; (3) generation of initial themes; (4) reviewing and developing themes; (5) refining, defining and naming themes; and (6) producing the report [30]. Open coding was performed, and an iterative reflexive process was used throughout the analysis and reporting phases. R. S., E. M. and J. F. familiarised themselves and open coded two transcripts each having first reviewed NPT constructs (Table 1). After review of the first two transcripts, R. S., E. M. and J. F. met to discuss codes to enhance reflexivity and depth of analysis.

R. S. then analysed all transcripts using open coding with highlighting and annotation functions in Microsoft Word (Seattle, USA). Coded data items were transferred from Word to Excel (Microsoft, Seattle, USA) with a spreadsheet set up for each interview, extracts numbered and columns created for iterative codes and reflexivity notes. This included a process of going back and forth between the transcripts, codes and themes to remain close to the data and iteratively review the meaning and develop themes.

In total, 986 extracts were coded. The coded extracts were then moved into individual spreadsheets after initial themes were developed manually in the first instance using differently coloured Post-it notes (3 M^©^) on a wall to cluster codes. The full research team (R. S., E. M., J. F., D. S.) met to further discuss codes, subthemes and themes after analysis of all transcripts by R. S., also to enhance reflexivity and refine themes consistent with RTA. Ten themes were initially generated and refined to 5 provisional themes before 2 were merged to create 4 final themes. K. A. contributed further to reflexivity and analysis during the reporting phase.

## Analysis^1^ (results)

**Fig. 1:**
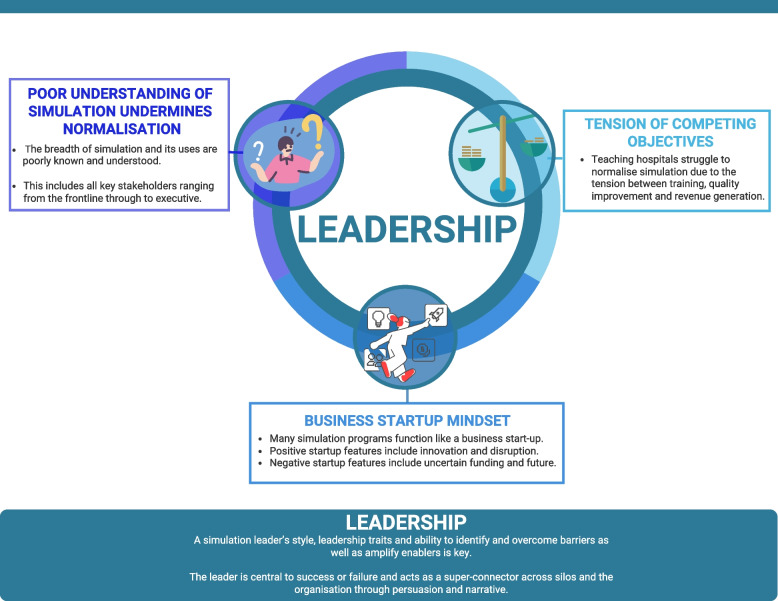
Infographic representation of interlinked themes

Four themes were generated from the data: (1) leadership, (2) business startup mindset, (3) poor understanding of simulation undermining normalisation and (4) tension of competing objectives. A graphical representation of the themes is provided in Fig. 1, and an overview of themes with characteristics is outlined in Table 2.

**Table 2:**
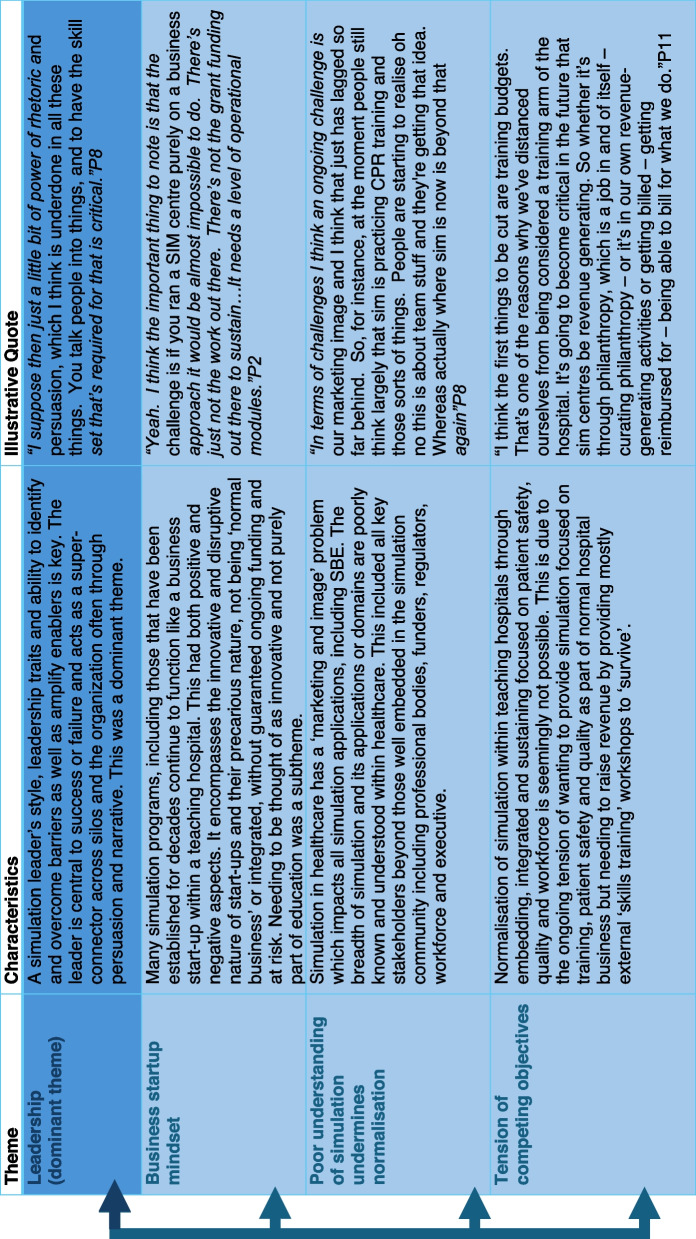
Overview of interlinked themes and their characteristics

Our data suggested a heightened focus on the relationships that influence decision-making by simulation leaders to bring about buy-in and commitment by executives, middle management and hospital workforce. It was clear these relationships were integral to a discrete programme becoming embedded within hospital operations. While SBE programmes may have become embedded, few had become integrated as a normal part of operational business, reflected in the survival language used by leaders. Interlinked themes represented how leaders experienced this process.

### Theme 1: Leadership

The importance of the leader and how they developed and used their social capital to network and influence others was a dominant theme, linked to all other themes. Participants described their influence as leaders and how their leadership traits were paramount for embedding SBE. Codes and subthemes related to ‘engagement of people’, the ‘sell’ and executive ‘buy-in’ and they as leaders influenced hospital subcultures to achieve normalisation.

I suppose then just a little bit of power of rhetoric and persuasion, which I think is underdone in all these things. You talk people into things, to have the skill set that’s required for that is critical. P8

Perceptions of ‘sub-cultures’ or ‘tribes’ within a teaching hospital influenced decisions about ‘who to play with’, including governance structures. This applied to not just healthcare professions (medicine, nursing, allied health) and specialty discipline cultures (like surgery and anaesthesia) but also operational groups like education, quality and safety, management and executive.

The data highlighted the relationships leaders’ developed to educate and influence others about simulation, and this finding particularly aligns with the NPT construct of ‘coherence’ or understanding (see Table 1). NPT contends that implementation is impeded when an intervention is not understood [6], making this an important finding. Sharing a clear vision of what simulation is and how it could address organisational needs was seen as vital. This was linked to theme 2 — business startup mindset and theme three — poor understanding of simulation undermines normalisation.

Find out what the mission of your hospital is from your CEO, and just align sim with that… Put yourself right in the middle of that. P11

It was seen as the role of able leaders to advocate for the purpose of simulation beyond procedural skill training and to explicate how simulation could address organisational priorities. There was also a sense from leaders that they needed to do the heavy lifting with regard to influencing all stakeholders, because simulation was not yet seen as integral to the organisation.

The other thing was having a champion and having somebody who – and it sounds like I’m blowing my own trumpet – somebody who was passionate in the face of most people. P5

The relationship building and influence of simulation leaders were central to all other themes. Their ability to amplify enablers, identify the needs and work across silos was vital to normalising simulation. Passion, tenacity and being strategic enabled these leaders to persist in the face of opposition or barriers, developing skills to overcome these through trial and error.

Evidence, experience and expertise were insufficient to effect change. Rather, knowing people and being known were vital. Constant reinforcement, through formal and informal channels to ‘bring people along’ P10, was viewed as essential, ‘I was trying to build bridges and partnerships within the organisation’ P3. Leaders’ abilities to influence change across reporting lines was integral to success.

This aligns with the Influence Without Authority leadership model first described in the 1980s by business management experts Cohen and Bradford [37, 38]. This model explains how those who do not have direct authority can encourage cooperation from those responsible for resources and funding as well as obtain buy-in from all stakeholders including middle management and executive. ‘Super-connectors’ is a term used to describe key informal influencers within an organisation. Three per cent of people in an organisation will potentially influence 85% of other people [39, 40], making super-connectors extremely influential in an organisation and responsible for most communication and change, particularly in times of uncertainty. In our study, it was clear that successful leaders were not just champions promoting SBE; they were super-connectors, able to influence others and amplify their programmes formally and informally.

### Theme 2: Business startup mindset

This theme encompasses simulation leaders’ descriptions of their experiences of embedding SBE programmes mirroring that of a ‘business startup’. Features of a startup include rapid adaptation to develop an innovative product or service, bring it to consumers and cement its place in the market, and at scale [41, 42]. During the startup phase, there is often uncertainty and disruption [42]. Startups are often, but not always, associated with something novel and innovative, and they necessarily defy the bounds of NPT.

Simulation leaders’ experiences reflected that simulation programs largely function like a ‘business startup’ within a teaching hospital related to uncertain funding and providing what is still considered by many an innovative ‘product’. Leaders described needing to be agile and strategic. This aligns with the disruptive nature of startups, and that they often progress rapidly and ‘fail fast’ — something that is usually not equated with large teaching hospitals — except in a pandemic.

So if I had to do it all over again I think I still probably would have moved quickly to be able to do things even before they had consensus adoption. P13

This linked to the dominant theme of leadership with a leader’s traits being integral to understand and work within an uncertain environment. Leaders described needing to reframe ‘what simulation is’ and be thought of as innovative, not purely for education.

Every other sim centre is thought of as an education arm – and certainly education is part of what we do… But we think of ourselves – and more importantly, the hospital thinks of us as a tool to solve mission-critical challenges for the hospital. P11

The discourse of ‘survival’ was strong and illuminated that normalisation of SBE was still a goal, rather than a reality. Most programmes were still in early phases or moving back to this phase due to external influences and uncertainty, particularly around funding and organisational buy-in. The meaning of this theme embraced that startups are not attempting to be ‘normal’; they yield benefits because they operate in a ‘unique’ way yet are also inherently precarious. Of note, 90% of business startups fail [41]. The unique benefits of a startup include being more likely to work at a rapid pace and pursue different types of opportunities while being agile and dynamic [41].

The unique benefits of a startup may indicate that while striving for normal would secure operations and funding, normal may be problematic and may impede innovation and buy-in. Startup benefits were offset by the risks related to uncertainty, particularly funding, as well as a demonstration of value through communication and evaluation. This relates to the NPT construct of reflexive monitoring (Table 1) and the importance of ongoing work required to ‘sell’ the mission as highlighted by Eller et al. [43].

Some simulation leaders noted there were times they lost influence, most often due to a change of hospital leadership and strategic direction or by not being able to show benefit. A loss of influence often resulted in derailing of simulation programmes, even if they felt near ‘normal’, contributing to the sense of them being precarious like a ‘business startup’. Reliance on influential leaders who are innovative and use persuasion (theme 1) may be both beneficial and risky [44].

### Theme 3: Poor understanding of simulation undermines normalisation

This theme reflects participants’ descriptions that the breadth of simulation is still not understood across healthcare. This lack of understanding extended from executive and management through to the frontline, regulating bodies and some in health professions education and those delivering SBE. Leaders described this being their own experience when they were new to simulation. This lack of understanding was seen to hold back the mission.

People see it as a fad, but I also think there’s a whole lot of people that don’t understand what simulation is. P3

While this research focused on SBE, leaders described using the breadth of simulation to obtain buy-in and engage hospital executives and key stakeholders largely related to solving key patient safety problems. They emphasised the importance of knowing ‘what simulation is’ beyond education because quality improvement (QI) and system change were key for patient safety. Leaders reported that this understanding influenced if a programme may be operationalised and of greater value.

Although people were viewing it under education the key is viewing it also under patient safety and outcomes. P2

The largest obstacle was needing to ‘sell’ and ‘educate’ others about using simulation for patient safety and system testing. The inclusion of simulation for QI, systems and equipment testing as well as to optimise teamwork and communication was viewed as powerful. While this mission was clear for simulation leaders as they matured, they perceived that others within healthcare and health professions education did not share this broader understanding of simulation.

An ongoing challenge is our marketing image and I think that just has lagged so far behind… at the moment people still think largely that sim is practicing CPR training…. People are starting to realise oh no this is about team stuff …. Whereas where sim is now is beyond that again. So, I think we’re going to be stuck with people thinking about dummies and practising…As sim evolves then it will inevitably involve more virtual reality, augmented reality and other, you know, and being in situ and things, people won’t really get it. P8

Leaders’ capacity to effectively communicate the breadth and benefits of simulation related to influence and persuasion (theme 1) and at times led to deliberate consultation with communications’ teams and other experts. Leaders particularly described their frustration when trying to progress simulation in situ for quality improvement and teamwork, beyond perceived ‘usual business’ of training. When this persuasion was done well, it elevated both SBE and simulation broadly.

I think it’s again – I feel this is kind of the reflection of the lack of understanding of what SIM is… P16

Simulation in healthcare was seen to have a ‘marketing and image problem’P8 which impacts all simulation uses, including SBE. This interlinked across themes, particularly with theme 1 and the concept of having a ‘clear vision and mission’ P10. The meaning of this theme connected to all four NPT constructs, particularly ‘coherence’ (Table 1) and the ‘communal specification’ component where there is a sense that everyone needs a shared view about an intervention’s purpose [45].

### Theme 4: Tension of competing objectives

Normalisation of simulation within teaching hospitals was difficult based on leaders’ experiences. This is due to the ongoing tension of wanting to provide both SBE and simulation for QI but needing to revenue raise by providing external ‘traditional SBE’ workshops to survive. The flip flop in purpose was seen to interfere with normalisation.

The challenge in all of that is that if you’re seen as a business unit there’s an expectation or there could be an expectation that you’re expected to raise everything. So you have to sort of think strategically about the way you account for the work that you do. So there’s the duality here. We supply service to the hospital at almost little to no cost and we also supply services to clients. The tension is how much work we can do in any one year that balances those two needs out. P2

While this theme related to other themes, particularly business startup mindset, the tension of competing objectives was itself dominant. Participant’s descriptions aligned with the NPT construct of reflexive monitoring, with ongoing appraisal of whether a programme could be adapted for the future. There was a shared understanding of both organisational and geopolitical influences relating to uncertainty, austerity and needing to find other funding sources. Some leaders described using research as a wheel for both legitimacy and sustainability via grant funding. There were significant pressures if funding was cut. Being viewed solely as part of education was detrimental versus being viewed as an essential part of QI and patient safety was beneficial.

The other barrier is where does education sit as a priority in the organisation? ….as soon as you have a cut in funding anywhere, education is always the first to go. P3

In striving for ‘normal’ leaders described, moving away from centre-based SBE and moving towards integration in hospitals. Thus, the goal in becoming normal was to be ‘integral to the institution’ P11 and ‘integrated into a clinician’s life at the clinician’s place’ P2.

Will we be ever come a society like – don’t laugh at me – like Star Trek, where there is no money and it’s purely driven by curiosity? That’s when you’ll have sustainability. While we have a society out there that’s driven by money, growth, it’s going to be quite – I think quite difficult. P3

The meaning of this theme encompasses the precariousness of SBE programmes, even those that were well-established. This largely related to changing geopolitical contexts and changes within hospitals influencing competing objectives.

## Discussion

SBE is not guaranteed to be a normal part of a teaching hospital based on the lived experience of simulation leaders in our study. Using the principles of NPT, ‘normal’ would mean SBE programmes are embedded, integrated and sustained, with a secure guaranteed budget, like any operational hospital unit. Leaders described this being the desired goal but difficult to achieve in practice. Our data found that embedding and integrating SBE are heavily dependent on the relationships formed by simulation leaders, and they benefitted from leveraging the use of simulation for QI and patient safety. However, we also found that the broad applicability of simulation (not just SBE) is not well understood, hampering implementation overall.

Although we sought to understand how simulation can be ‘normal’, our data demonstrates that there were some benefits to simulation being seen as innovative and novel, like a startup. Simulation programmes may benefit from a startup mindset to be adaptive and responsive to organisational needs. However, the challenge is the juxtaposition of innovation, disruption and something novel with the desire and need for sustained and secure funding, resources and established governance which come with being integrated into normal operations.

Our analysis found that this desired and necessary aim of being a normal part of operations risks a programme becoming stagnant and irrelevant. Our data highlighted the importance of a broader and coherent understanding of all uses of simulation, particularly for QI and patient safety, to be responsive to a hospital’s needs and consequently embed, integrate and sustain simulation for all purposes. Based on our data, we expand on a key observation with reference to the literature — that simulation has ‘a marketing problem’ due in part being poorly known and understood. The experience of our participants was that this is a significant impediment to normalisation.

Leaders’ experiences challenged us to think beyond SBE and what people in healthcare know and understand about simulation broadly. Our data demonstrated that leaders needed to repeatedly use persuasion and rhetoric to explain ‘what is simulation’, including SBE. The lack of understanding of simulation was encapsulated best by one participant in our study noting that many do not know what simulation is ‘beyond CPR on a manikin’. This aligns most to the NPT construct of ‘coherence’ which involves understanding, internalising and applying a method to solve a problem [15]. Thus, NPT maintains that a lack of knowledge and understanding may impede implementation [22]. Ferguson et al. also found that ‘participants reported that their organisation’s leaders, colleagues and students often had poor knowledge of what simulation was and the potential benefits for practice’ [6].

Notably, within our data, lack of coherence of what simulation is in healthcare was described as a ‘marketing and image problem’ heavily reliant on the leader to do the ‘educative work’ and promotion within their hospital. This was meaningful because leaders described simulation for patient safety and QI being influential for normalisation and long-term buy-in for all uses of simulation, including SBE. As Brazil et al. highlighted in 2019, while SBE is established, simulation as a QI tool is still nascent [46]. While it is nascent, leaders highlighted it as the most impactful. A shared understanding of the breadth of simulation across all healthcare — including executives, regulatory and funding bodies is needed to move simulation beyond being an ‘add-on’.

Simulation may have a marketing problem because it is not one thing. It is hard to communicate something that is a multifaceted tool with many uses versus communicating what underpins decisions to use that tool and why it is used. Although simulation has been categorised into four domains, (T1) education in the lab, (T2) patient care practices, (T3) patient outcomes and (T4) collateral educational effects [10], these are not known beyond the simulation community. Victoria Brazil’s description of translational simulation addresses this lack of coherence by explaining ‘why simulation’ [47]. Translational simulation encapsulates how simulation may be viewed based on function related to health service priorities, as a tool for education, QI and systems testing [47].

Translational simulation is particularly valuable terminology to communicate with grant, research and government bodies focused on translational research. The meaning of ‘translational research’ is broadly understood within healthcare as being from bench-to-bedside and bedside-to-community [10]. This shared understanding for scientists, clinicians, funders and the community has provided coherence and mission that we as a simulation community can leverage, if we ensure the full breadth of ‘simulation’ in healthcare is also understood.

Our data supports that the meaning of ‘simulation’ is still not broadly understood. Our findings indicate that a considerable gap in the understanding of the breadth of simulation exists across healthcare professions, including for many healthcare educators not immersed in simulation. This limited understanding extends to hospital and government leaders, regulatory bodies and funders. Crucially, the narrow perception of simulation’s scope as SBE for basic procedures like ‘CPR on a manikin’ may be undermining its acceptance, financing and practical implementation in hospital settings.

We contend that both implementation science and communication principles could support building coherence and a broad understanding of simulation which plays a pivotal role in normalisation. Simulation being ‘known’ and positioned for education, QI and patient safety is perhaps more important than what simulation is or is not. This aligns with needing to have a clear mission to connect simulation and QI as described by Brazil et al. [46]. Our data demonstrate there remains a gap in that mission being understood, and the theme ‘a business startup mindset’ may provide some guidance for addressing this gap.

Healthcare and the modern marketplace are environments with enormous amounts of information [48, 49]. Business startups often place inadequate attention on establishing their ‘brand’ by prioritising financial and operational concerns [41, 50, 51]. This is mirrored in our data. Successful companies like Apple Inc. have overcome such marketing issues by focusing on image and corporate position through storytelling and mission — over product [48]. The mission of simulation is patient safety and systems improvement, whether for education or QI [8]. The simulation community collectively adopting an approach of clearly communicating this mission may address the ‘marketing problem’. Reframing simulation as a QI and safety mindset, not just a tool nor a technology, may be one way to communicate this mission and ensure ‘simulation’ is well known and understood, ‘beyond CPR on a manikin’.

Based on our findings and linked to the NPT construct of coherence, we invite the simulation community to consider how to broadly communicate what simulation means and why it is needed. This echoes Gaba’s 2004 vision that ‘the simulation community must educate the public and the implementing agencies on the vision of improved patient safety using the tool of simulation’ ([8] p.i8). Our study strongly suggests that SBE is far from normalised in teaching hospitals. A focus on communication and promotion of a coherent identity for simulation is recommended. An approach of promoting translational simulation terminology and marketing simulation as a patient safety and systems improvement mindset would support the mission of simulation becoming broadly known and understood at all levels of healthcare. This could support simulation being equitably and sustainably embedded and integrated.

### Strengths and limitations

The lead researcher’s evolution in becoming a leader in simulation during the course of this work led to a combined social and experiential constructivist worldview. This experiential and Big Q approach to the research may mean that other researchers would come to other conclusions with our data. We believe this approach strengthens the work making it more applicable than if someone without lived experience of normalising SBE in a teaching hospital had conducted this research. We believe NPT as an analytical lens strengthens the work, alerting the research team to dimensions of ‘embedding’ practices that we may not have noticed without this priming.

Our analysis also highlighted the benefits of simulation remaining novel within an organisation. Being seen as ‘novel’ versus normal’ was at times advantageous in attracting interest from stakeholders, generating media attention, accessing innovation grants and gaining ‘kudos’ for being novel and innovative’. The use of NPT to inform the analysis helped us to see how simulation could be normalised but paradoxically challenged the concept that ‘normal’ is the desired end state. We focused on high-income English-speaking countries only, meaning leaders’ experiences in other contexts may be very different.

### Future research and practical implications

Understanding the experiences of middle management, quality and safety and executives and those in regulatory bodies of embedding, integrating and sustaining simulation in teaching hospitals is an underexplored area. Further research on how all stakeholders view simulation within a teaching hospital is warranted. Of note, in our data set, we saw a strong thread relating to ‘startups’ which are defined as such in the business literature because of their unique properties. This challenged us to consider whether ‘normalising’ a practice is always the desired end goal. Participants did for example describe the ‘pay offs’ in leading a ‘novel’ service or programme that was yet to be routine. Investigating further the intersection between a startup phase and normalisation is another area for future research.

Exploring the business and entrepreneurship literature as well as partnering with those engaged in startups and the business world may be one path to consider in addition to collaborating with implementation science and practice experts. Further research on how these leaders achieve buy-in from others is needed as well as exploration of how to teach and develop these skills in simulation teams. A qualitative case-series approach, including observational and ethnographic data to extend the understanding beyond a self-reported account by leaders, is recommended [52]. Our data also support teaching of leadership skills to current and future leaders in simulation.

Understanding the experience of embedding simulation in low- and middle-income countries and high-income non-English-speaking countries is needed for a comprehensive understanding of the implementation of simulation globally. As further work in this area may address the sporadic and unequal distribution of simulation globally, these areas are a rich source of data for future research.

## Conclusion

Using RTA sensitised by NPT, we have generated four interlinked themes and identified some of the shared experiences of healthcare simulation leaders of normalising an SBE programme in a teaching hospital. There was a heightened focus on leadership and relationships. Participants spoke of trying to normalise simulation for patient safety, but there was also a strong sense that leaders need to be agile and innovative, indicating there may be some benefit in a startup mindset. There is therefore a tension of being adaptive and responsive to organisational needs with being a normal part of operations. Leadership, change management and entrepreneurship in addition to implementation science may all contribute towards normalising simulation in teaching hospitals without losing a business startup mindset. This study has highlighted that a shared understanding embracing the purpose and breadth of simulation is a prerequisite for embedding, integrating and sustaining simulation in teaching hospitals. An approach of marketing simulation including SBE as a patient safety and systems improvement mindset, not just a technique nor technology, may assist towards it being broadly understood.

## Data Availability

Supplementary material including plain language statement, consent and interview guide is available on request. The datasets generated and analysed during the current study are not publicly available but are available from the corresponding author on reasonable request.

## Abbreviations

ASSH: Australian Simulation Society in Healthcare
CPR: Cardiopulmonary resuscitation
IMRD: Introduction, methods, results, and discussion
HEAG: Human Ethics Advisory Group
NPT: Normalisation process theory
P: Participant
PDF: Portable document format
QI: Quality improvement
RTA: Reflexive thematic analysis
SBE: Simulation-based education
SSH: Society for Simulation in Healthcare
USA: United States of America

## Footnotes

1 In keeping with RTA and a Big Q, stance analysis will incorporate literature and researcher experience. This may read more like a discussion to those familiar with a traditional quantitative or small q IMRD framework of reporting. Elements of a traditional discussion are woven through this type of analysis reporting style and a general discussion follows.
